# An internet-based self-help intervention for people with psychological distress due to COVID-19: study protocol for a randomized controlled trial

**DOI:** 10.1186/s13063-021-05089-9

**Published:** 2021-03-01

**Authors:** Noemi Anja Brog, Julia Katharina Hegy, Thomas Berger, Hansjörg Znoj

**Affiliations:** Department of Psychology, University of Bern, Fabrikstrasse 8, 3012 Bern, Switzerland

**Keywords:** Anxiety, Coronavirus, COVID-19, Depression, Internet-based self-help, Psychological distress, Randomized controlled trial, Stress

## Abstract

**Background:**

The coronavirus-19 (COVID-19) has reached pandemic status and is affecting countries all over the world. The COVID-19 pandemic is accompanied by various stressors that require adjustment in everyday life and possibly changes in personal future prospects. While some individuals cope well with these challenges, some develop psychological distress including depressive symptoms, anxiety, or stress. Internet-based self-help interventions have proven to be effective in the treatment of various mental disorders such as depression and anxiety. Based on that, we developed an internet-based self-help program for individuals with psychological distress due to the situation surrounding the COVID-19 pandemic. The 3-week self-help program consists of 6 modules comprising texts, videos, figures, and exercises. Participants can request guidance within the self-help program (guidance on demand). The primary aim of this study is to evaluate the efficacy and feasibility of the self-help program compared to a waiting control condition.

**Methods:**

The design is a parallel group randomized controlled trial. Participants are allocated to a 3-week self-help intervention plus care as usual or a 3-week waiting period with only care as usual. There are follow-ups after 6 weeks and 18 weeks. At least 80 participants with COVID-19 pandemic related psychological distress will be recruited. Primary outcome are depressive symptoms. Secondary outcomes include anxiety and chronic stress, suicidal experiences and behavior, health-related quality of life, generalized optimism and pessimism, embitterment, optimistic self-beliefs, emotion regulation skills, loneliness, resilience, and the satisfaction with and usability of the self-help program.

**Discussion:**

To the best of our knowledge, this is one of the first studies investigating the efficacy of an internet-based self-help program for psychological distress due to the situation surrounding the COVID-19 pandemic. Thus, the results of this study may give further insight into the use of internet-based self-help programs in pandemic-related psychological distress.

**Trial registration:**

ClinicalTrials.gov NCT04380909. Retrospectively registered on 8 May 2020.

## Administrative information

The order of the items has been modified to group similar items (see http://www.equator-network.org/reporting-guidelines/spirit-2013-statement-defining-standard-protocol-items-for-clinical-trials/).

**Table:** 

Title {1}	An internet-based self-help intervention for people with psychological distress due to COVID-19: study protocol for a randomized controlled trial.
Trial registration {2a and 2b}.	ClinicalTrials.gov, NCT04380909, Retrospectively registered on 8 May 2020
Protocol version {3}	2021 January 28, Version 3
Funding {4}	The study is entirely funded by the University of Bern.
Author details {5a}	NB^1^, JH^1^, TB^1^, and HZ^1^ ^1^Department of Psychology, University of Bern
Name and contact information for the trial sponsor {5b}	Trial Sponsor: University of Bern, Contact name: Prof. Dr. HZ, Address: Fabrikstrasse 8, 3012 Bern, Switzerland, Email: hansjoerg.znoj@psy.unibe.ch
Role of sponsor {5c}	The funding body (University of Bern) played no role in the design of the study, the collection, analysis and interpretation of data or in writing the manuscript. However, the trial sponsor is the principal investigator of the study (sponsor investigator).

## Introduction

### Background and rationale {6a}

The coronavirus-19 (COVID-19) has reached pandemic status and is affecting countries all over the world. Health systems are facing major challenges: In addition to the risks for physical health, the COVID-19 pandemic also represents a burden for mental health [1]. Pandemic-related stressors such as quarantine, social isolation/distancing, unemployment, financial losses, caregiver stress, and confrontation with illness and death can have a negative impact on mental health [1]. For example, in a study on the severe acute respiratory syndrome (SARS) pandemic, approximately 40% of the study population experienced increased stress related to work, finances and family and 16% showed signs of posttraumatic stress [2]. Preliminary research on the psychological impact of the COVID-19 pandemic indicates increased levels of psychological distress in the general population [3, 4]. Symptoms of anxiety, depression, and self-reported stress are suggested psychological reactions to the COVID-19 pandemic [5, 6].

Although pandemics comprise a multitude of stressors that may strain mental health, not everybody is experiencing psychological distress in response. Moreover, individuals might differ in the amount and kind of stressors they are exposed to, and therefore, some individuals might be at higher risk for mental health problems [7]. Some of the stressors that occur during a pandemic can be considered critical life events (e.g., death of loved ones and job loss) and require adjustment to changed life circumstances [8]. A lack of adjustment can lead to psychological distress, for example expressed in a change of one’s psychological condition. This can include experiencing depressive and anxiety symptoms [9]. Furthermore, maladaptive adjustment to critical life events might eventually lead to full-blown mental disorders like adjustment disorders (AjD) or depression [10–12].

Some recommendations for interventions targeting psychological distress due to the COVID-19 pandemic have been made: Firstly, cognitive behavioral therapy (CBT), in particular the restructuring of thought patterns and cognitive thinking traps, as well as activity planning and relaxation techniques are considered suitable interventions [6, 13]. Secondly, digital aids such as internet-based self-help interventions are encouraged, as they do not require physical contact and are easily scalable [6, 14, 15].

The efficacy of internet-based self-help interventions for various psychological problems is established [16, 17]. However, internet-based interventions can differ in their design, especially in the degree of therapist support that they offer. While some internet-based interventions offer contact with a therapist (guided self-help) other interventions are completely automated (unguided self-help). Moreover, guided self-help interventions can differ in the intensity of provided contact. On the one hand, guided self-help programs yield higher effect-sizes and have higher retention rates than unguided self-help programs [18, 19]. On the other hand, unguided self-help programs have the advantage that they are less costly and better scalable [20]. One promising approach, possibly combining the benefits of both guided and unguided self-help programs, is the use of guidance on demand [21]. Guidance on demand implies that support from a therapist is only established when requested by a participant. An internet-based self-help program for increased self-criticism with guidance on demand showed promising results [22]. Nonetheless, an internet-based self-help program for symptoms of anxiety and/or depression based on problem-solving therapy with guidance on demand had the same effect as the unguided version of the same program [23]. Likewise, an internet-based self-help program for tinnitus-related distress based on CBT with guidance on demand did not differ in its effectiveness from the unguided version of that program [24].

To the best of our knowledge, there is no study that has evaluated an internet-based self-help intervention for psychological distress due to COVID-19 in the general population yet. However, an internet-based self-help intervention for patients diagnosed with COVID-19 experiencing psychological distress has been evaluated in a small randomized controlled trial (RCT) [25]. The internet-based self-help intervention consisted of audio-recorded instructions focusing on relaxation, self-care, and a rising sense of security, which were uploaded online. Over a 2-week period, participants in the intervention group listened to the instructions via their mobile phone and performed a daily task, which took about 50 min. The intervention addressed COVID-19 patients with mild-to-moderate depression and/or anxiety symptoms. The average age of the 26 participants was 44.7 years; 62% were male and 38% were female. Ninety-two percent of the participants experienced at least mild depression symptoms and 62% experienced at least mild anxiety symptoms. Participants in the intervention group showed a significant reduction in depression and anxiety symptoms compared to the control group [25] .

Against this background, we developed an internet-based self-help intervention with guidance on demand called ROCO. This intervention specifically addresses individuals experiencing psychological distress due to the COVID-19 pandemic. Hence, the study aims to evaluate the efficacy and feasibility of the internet-based self-help program ROCO for people with psychological distress due to the COVID-19 pandemic.

### Objectives {7}

The specific objectives of the study are:

1. To evaluate the effects of the internet-based self-help program compared with a waiting control condition on:
  The primary outcome depressive symptoms
  Secondary outcomes such as anxiety and stress symptoms, well-being, embitterment, and loneliness
2. To evaluate the acceptance and user-friendliness of the internet-based self-help program and drawing conclusions for further developments of the program.
3. To exploratory search for predictors, moderators, and mediators for the efficacy of the program:
  e.g., optimism, age, severity of depressive symptoms, and frequency of use of the program

### Trial design {8}

**Fig. 1:**
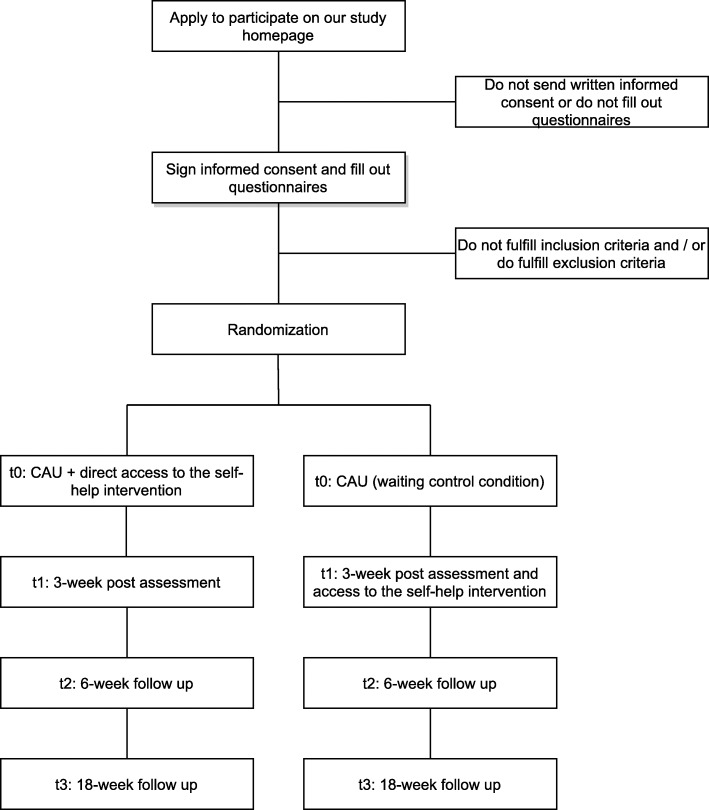
Flowchart of the study design

The study is a parallel group RCT comparing an internet-based self-help intervention combined with care as usual (CAU) to a waiting control condition with only CAU. The study flowchart is displayed in Fig. 1. Participants in the waiting control condition receive access to the intervention 3 weeks after the baseline questionnaire. Eligible participants are randomly allocated to one of the two conditions in a 1:1 allocation ratio.

The aim of the RCT is to show the superiority of the internet-based self-help intervention combined with CAU to only CAU at the 3-week post assessment. Additionally, there will be 2 follow-up measurements after 6, respectively 18 weeks after the baseline questionnaire. Since at the time of the follow-up measurements both groups have used the internet-based self-help intervention, the groups can no longer be compared. However, we use these follow-up measurements to assess the sustainability of potential treatment gains, i.e., to examine whether the short-term effects of the internet-based self-help intervention are maintained within groups. In addition, the follow-up measurements will be used to explore predictors of the sustainability of potential treatment effects.

## Methods: participants, interventions, and outcomes

### Study setting {9}

The single study center is located at the University of Bern, Switzerland. All data is collected online using questionnaires programmed in Qualtrics [26]. Data is collected in German-speaking areas (i.e., Switzerland, Germany, Austria, and Liechtenstein).

### Eligibility criteria {10}

All interested persons must provide full written informed consent and are required to complete a baseline-screening questionnaire prior to randomization to assess eligibility.

Inclusion criteria are:

1. To be at least 18 years old
2. To exceed a cutoff value of 4 points on the brief Patient Health Questionnaire (PHQ-9) [27]
3. To be able to specify an emergency address in the event of an acute crisis
4. To have access to the internet
5. To understand and master the German language to the degree that one understands the content and instructions of the study

Exclusion criteria are:

1. The presence of suicidal tendencies (score ≥ 8 on the Suicide Behavior Questionnaire Revised (SBQ-R) [28]
2. A known diagnosis of a psychotic or bipolar disorder

## Who will take informed consent? {26a}

Individuals interested in participating in the study can provide their e-mail address on the study homepage. Subsequently, they receive an e-mail with the detailed study information and the informed consent form. They are also asked to watch a video on the study homepage in which the study information is explained orally by the principal investigator. Individuals have the possibility to ask the study team questions about the study via e-mail. Written informed consent is obtained from individuals willing to participate in the study by the Principal Investigator.

### Additional consent provisions for collection and use of participant data and biological specimens {26b}

Not applicable as no ancillary studies are performed.

## Interventions

### Explanation for the choice of comparators {6b}

The efficacy of the intervention is to be established. This is why we have chosen a waiting control condition as comparator. However, both the participants in the waiting control condition as well as in the intervention condition receive CAU, whereby CAU can range from no treatment at all to psychotherapy and/or drug therapy. Participants in the waiting control condition receive access to the internet-based self-help program after a waiting period of 3 weeks. We decided to give participants in the waiting control condition access to the program after only 3 weeks since, due to the pressing situation surrounding the COVID-19 pandemic, we wanted to give all participants access to psychological support as fast as possible. However, this has the implication that only short-term effects of the intervention can be assessed.

### Intervention description {11a}

The intervention is a 3-week internet-based self-help program with guidance on demand called ROCO. The self-help program consists of 6 thematic modules including texts, videos, graphics, exercises, and for each module a weekly task. The 6 thematic modules are supplemented by an introduction and a conclusion. For a detailed description, see Table 1. Furthermore, the self-help program comprises a page with information on what to do in an acute crisis, including a list with emergency contacts, as well as a page named *Toolbox,* where the weekly tasks are listed. Participants also can track their symptoms on a page named *Mood-Tracker*.

**Table 1:** Outline of the content of the internet-based self-help program ROCO

Introduction	Information about the self-help program
1. Identifying consequences and challenges	Information about psychological distress/adjustment problems due to the COVID-19 pandemic, assessment of the current state (bodily sensations, positive and negative feelings), resource-oriented weekly task
2. Understanding own feelings	Information about feelings such as anxiety, helplessness, anger, sense of shame and sadness, strategies to cope with these feelings, acceptance-oriented weekly task
3. Changing the perspective	Information about the influence of thoughts, automatic thoughts, rumination and irrational beliefs, exercises to challenge own thinking patterns, weekly task on rumination
4. Strengthening resilience	Information about resilience and three possible ways of gaining resilience, namely coping, joie de vivre and optimism, exercises to promote these, resource-oriented weekly task
5. Finding rest	Information about sleep, sleep hygiene and relaxation techniques, progressive muscle relaxation as a weekly task
6. Taking care of oneself	Information about the concept of posttraumatic growth and the importance of pleasure, exercises of gratitude and mindfulness, resource-oriented weekly task
Conclusion	Information about the importance of practicing and transferring what has been learnt to daily life

Participants are encouraged to work through two of the 6 thematic modules per week. One module takes between 40 to 80 min to complete. However, participants can determine the timing and order of the modules themselves. The first module includes information about possible psychological consequences and challenges concerning the situation surrounding COVID-19. In the second module, participants receive information concerning ways to deal with difficult feelings that may arise due to the current situation. The third module focuses on restructuring thought patterns and cognitive thinking traps and the fourth module on promoting resilience and coping skills. The fifth module consists of information about sleep hygiene and relaxation techniques. Finally, the last module addresses self-care and personal growth.

As the self-help program offers guidance on demand, participants have the possibility to contact a psychologist, but there is no scheduled contact per se. Participants can require guidance via chat function in the self-help program. They are informed that a psychologist will answer their request within 3 working days. Otherwise, the self-help program is unguided.

### Criteria for discontinuing or modifying allocated interventions {11b}

Since internet-based self-help is not suited as a treatment for acute suicidality, participants reporting an acute crisis during treatment are referred to an appropriate treatment. This will be recorded and reported as an adverse event.

### Strategies to improve adherence to interventions {11c}

Participants have the possibility to enable reminders within the self-help program. They can choose whether the reminder is sent via e-mail or text message after a certain time of inactivity. In the reminder, participants are encouraged to log into the self-help program again. We have further adopted a guidance on demand approach, since some form of support appears to increase adherence [19].

### Relevant concomitant care permitted or prohibited during the trial {11d}

Participants receiving the intervention, as well as participants in the waiting control condition are allowed to start any concomitant treatment at any time during the trial. However, participants must indicate at each measurement time whether they use concomitant psychological or psychiatric treatment (e.g., psychotherapy or drug therapy).

### Provisions for post-trial care {30}

The University of Bern will provide insurance for any harm suffered as a result from this trial.

### Outcomes {12}

**Fig. 2:**
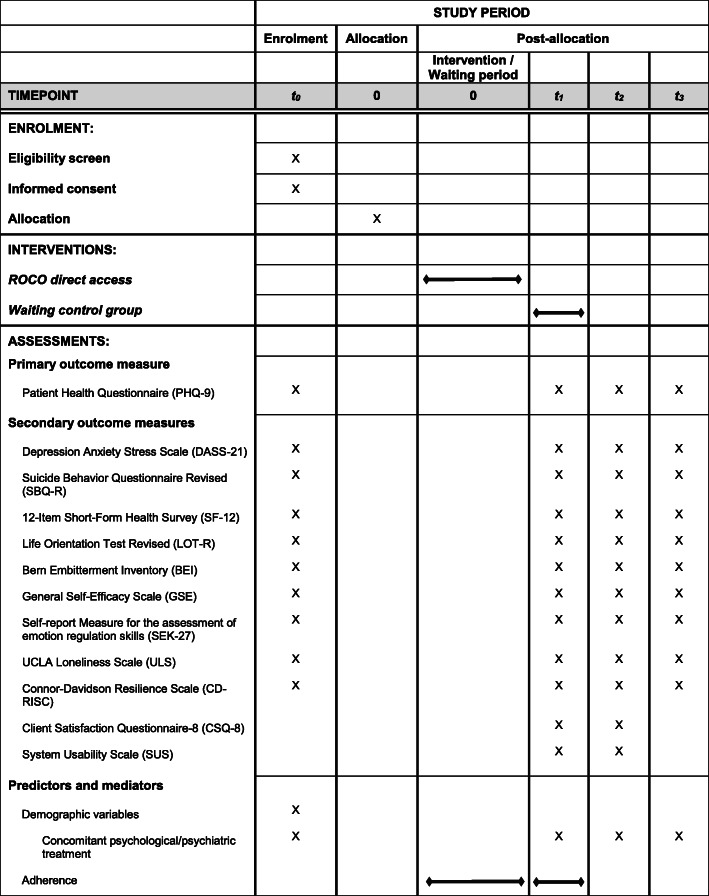
SPIRIT figure, schedule of enrolment, interventions, and assessments

All assessments are carried out online via self-observation questionnaires. The baseline measurement is at *t*_*0*_, the post-measurement *t*_*1*_ is at 3 weeks, the first follow-up measurement *t*_*2*_ is at 6 weeks, and the second follow-up measurement *t*_3_ is at 18 weeks after the baseline. Validated German versions of the questionnaires are used. For an overview of all outcome measures and corresponding measurement time points, see Fig. 2.

#### Primary outcome measure

##### Patient Health Questionnaire (PHQ-9)

The primary outcome measure is the score of the PHQ-9 [27]. The PHQ-9 is a 9-item measure assessing the severity of depressive symptoms. All 9 DSM-IV criteria for depression are scored on a scale from 0 = not at all to 5 = nearly every day. A score of 5 represents a mild depression, a score of 10 a moderate depression, a score of 15 a moderately severe depression, and a score of 20 a severe depression [29]. The PHQ-9 showed good internal consistency (Cronbach’s alpha between 0.86 and 0.89) [30, 31].

#### Secondary outcome measures

##### Depression Anxiety Stress Scale (DASS-21)

The DASS-21 is a short-form of the DASS and is used to assess depressive mood, anxiety, and chronic stress during the past week [32]. The DASS-21 consists of 21 items which are answered on a scale from 0 = did not apply to me at all to 3 = applied to me very much or most of the time. The internal consistencies of the scores for depressive mood, for anxiety, and for chronic stress (Cronbach’s alpha = 0.88, 0.76 and 0.86) lie between satisfactory and good [33].

##### Suicide Behavior Questionnaire Revised (SBQ-R)

The SBQ-R assesses suicidal experiences and behavior [28]. The SBQ-R consists of 4 items which are not scaled equally. A total score of the 4 items is calculated. The total score can range from 3 to 18 whereas a score greater than or equal to 8 is considered the most useful cutoff score for suicide risk in a clinical sample [28]. This SBQ-R cutoff is also used as an indication for suicidal tendencies (safety outcome). The internal consistency of the SBQ-R is satisfactory (Cronbach’s alpha = 0.72) [34].

##### 12-Item Short-Form Health Survey (SF-12)

The SF-12 assesses health-related quality of life and is the short version of the Medical Outcomes Study 36-Item Short-Form Health Survey [35]. The SF-12 consists of 12 items with varying answer format. There are two versions of the SF-12, one assessing the health-related quality of life over the past week and one assessing it over the past 4 weeks. In this study, the latter is used. From the 12 items of the SF-12, a Physical Component Score and a Mental Component Score can be calculated. The internal consistency of the subscales exceeds the recommended Cronbach’s alpha level of 0.70 [36] .

##### Life Orientation Test Revised (LOT-R)

The LOT-R is a 10-item scale assessing generalized optimism and pessimism [37]. The items are answered on a scale from 0 = strongly disagree to 4 = strongly agree. Three items form the score for pessimism and 3 items the score for optimism, whereas 4 items are unscored as they are filler items. The internal consistency is satisfactory with a Cronbach’s alpha of 0.69 for optimism and 0.68 for pessimism [38].

##### Bern Embitterment Inventory (BEI)

The BEI is an 18-item questionnaire assessing embitterment, whereby embitterment can be understood as the feeling of being disadvantaged by others and fate [39, 40]. The items are answered on a scale from 0 = I do not agree to 4 = I agree. The internal consistency for the total embitterment score is good (Cronbach’s alpha 0.89) [39]. In this study, the 6-item short version of the BEI is used [41].

##### General Self-Efficacy Scale (GSE)

The GSE is a 10-item questionnaire assessing optimistic self-beliefs [42]. The items are answered on a scale from 1 = not at all true to 4 = exactly true. The internal consistency (Cronbach’s alpha) for the total score ranges between .76 and .90 [42].

##### Self-report measure for the assessment of emotion regulation skills (SEK-27)

The SEK-27 assesses adaptive ways of coping with negative emotions [43]. The 27 items are answered on a scale from 0 = never to 4 = (almost) always. Two versions of the SEK-27 are available: a trait version assessing the coping with negative emotions in general and a prolonged state version assessing the coping with negative emotions over the last week. In this study, the latter is used. A total scale as well as the subscales attention, bodily awareness, clarity, understanding, regulation, acceptance, resilience, self-support, and goal-oriented readiness for confrontation can be formed. The total scale of the prolonged state version has an excellent internal consistency (Cronbach’s alpha = 0.90). The internal consistency of the subscales of the prolonged state version ranges from 0.72 to 0.81 [44].

##### UCLA Loneliness Scale (ULS)

The ULS is a measure assessing one’s subjective feeling of loneliness [45]. The items are answered on a scale from 1 = never to 4 = often. The original version of the ULS consists of 20 items and has an internal consistency (Cronbach’s alpha) ranging from 0.82 to 0.92 [45]. In this study, a 9-item version of the ULS is used.

##### Connor-Davidson Resilience Scale (CD-RISC)

The CD-RISC assesses resilience [46]. Items are answered on a scale from 0 = not true at all to 4 = true nearly all of the time. In this study, the 10-item version of the CD-RISC is used. The 10-item version has a good internal consistency (Cronbach’s alpha) of 0.84 [47].

##### Client Satisfaction Questionnaire-8 (CSQ-8)

The CSQ-8 assesses the satisfaction of the participants with the intervention [48]. The CSQ-8 consists of 8 items which are answered on a scale from 1 = poor to 4 = excellent. Since the CSQ-8 measures the satisfaction with the intervention, it can only be used after the intervention phase. THE CSQ-8 has an excellent internal consistency (Cronbach’s alpha) ranging from 0.87 to 0.93 [49].

##### System Usability Scale (SUS)

The SUS is used to assess the usability of a system such as mobile devices, websites and applications [50]. The 10 items of the SUS are answered on a scale from 1 = strongly disagree to 5 = strongly agree. A score between 0 and 100 can be calculated, indicating the usability of a system, in this case the internet-based self-help program. Since the SUS measures the system usability of the internet-based self-help program, it can only be used after the intervention phase. The English version of the SUS has an excellent internal consistency (Cronbach’s alpha) ranging from = 0.91 to 0.92 [51, 52].

#### Predictors and moderators

##### Demographic variables

Demographic variables include sex, age, country of residence, civil status, housing situation, current childcare situation, education, employment situation (before and during COVID-19 pandemic), income (before and during COVID-19 pandemic), current everyday working life, psychiatric medical history, concomitant psychological/psychiatric treatment, and COVID-19-specific questions (e.g., belonging to a risk group, own illness or instances of deceased family members due to the pandemic).

##### Adherence

The intensity and frequency of use of the self-help program is measured by indicators collected within the self-help program such as percentage of accessed pages or number of logins.

### Participant timeline {13}

(Figure 2).

### Sample size {14}

To specify the sample size needed for the different analyses, we conducted a power analysis based on a probability level of 0.05 and a power of 0.80 with G*Power [53] for a repeated-measures ANOVA with a within-between-interaction. To test the efficacy of the self-help program compared to the control condition, we expected a small-to-medium between group effect size of *d* = 0.35 and a correlation between the factors of at least *r* = 0.4. The expected effect size is based on the results of meta-analyses on the effectiveness of unguided internet-based self-help programs targeting depressive symptoms [17, 54]. We decided to base the expected effect size on unguided internet-based self-help programs as it is yet to be determined if a guidance on demand approach yields higher effect sizes than unguided self-help [23, 24] . Power analysis indicated a necessary sample size of 80 individuals. In consideration of a possible attrition rate between 5.4 and 45.5% at post-assessment, we aim to recruit between 80 and 120 participants at baseline [17].

### Recruitment {15}

Participants are recruited from the general population via the study web page. This study web page is advertised on various websites, internet forums and social media. The study web page includes information about the self-help program and the study. People interested in participating can leave their e-mail address on the study homepage and will then be sent the detailed participant information per e-mail.

## Assignment of interventions: allocation

### Sequence generation {16a}

Eligible participants will be randomly allocated to either the intervention or the waiting control condition with a 1:1 allocation ratio as per a computer generated randomization schedule using randomly permuted block sizes by Randomization.com [55].

### Concealment mechanism {16b}

The allocation schedule was generated by an independent researcher and is unknown to the investigators. Allocation takes place after the baseline assessment has been completed. Since the allocated condition is not known until the interested individual has been recruited into the trial, allocation concealment is ensured.

### Implementation {16c}

All interested individuals who give written informed consent for participation and who fulfill all the inclusion criteria and none of the exclusion criteria will be randomized. Staff members responsible for recruitment and data management will ask the independent researcher to randomize respective individuals. In return, the independent researcher informs the staff members per e-mail about the allocation. Finally, the staff members inform the individual about the assigned condition per e-mail.

## Assignment of interventions: blinding

### Who will be blinded {17a}

The staff members are not blinded to the allocation. However, all assessments are performed online with self-report questionnaires. Since participants either receive direct access to the self-help program or have a waiting period, participants are neither blinded to their allocation.

### Procedure for unblinding if needed {17b}

Not applicable since no blinding is performed.

## Data collection and management

### Plans for assessment and collection of outcomes {18a}

All data is assessed online by means of questionnaires programmed in Qualtrics [26]. In addition, data on the use of the self-help program (e.g., number of logins or processed pages) is collected within the self-help program.

### Plans to promote participant retention and complete follow-up {18b}

The participants are asked by e-mail to complete the online questionnaires. If participants fail to complete a questionnaire, they will be reminded by e-mail to do so: for post measurement, they will be reminded after 5 and 10 days and for follow-up measurements after 7 and 14 days. All participants are asked to complete the online questionnaire at each point of measurement, regardless of protocol adherence or any previously uncompleted online questionnaires.

### Data management {19}

Data quality is ensured through several mechanisms, including referential data rules, valid values, range checks, and consistency checks. The option to choose a value from a list of valid codes and a description of the meaning of the code will be available where applicable. Checks are applied at the time of data entry into a specific field. All data collected is stored on a firewall-encrypted back-upped server of the University of Bern with strictly regulated access only for researchers directly involved in the study.

### Confidentiality {27}

All data concerning participant information will be stored in locked file cabinets only accessible for staff members. All collected data will only be traceable by a code. All files containing names or other personal identifiers, such as the informed consent forms, will be stored separately from data containing this code number.

### Plans for collection, laboratory evaluation, and storage of biological specimens for genetic or molecular analysis in this trial/future use {33}

Not applicable since no biological specimens are used.

## Statistical methods

### Statistical methods for primary and secondary outcomes {20a}

We will use linear mixed models with time (pre versus post-intervention measures) as a within-group-factor and study condition (immediate access versus control condition) as a between-group-factor to evaluate the efficacy of the internet-based self-help intervention. This primary analysis will be performed using the data from the baseline and the 3-week post assessment. To analyze the stability of the short-term effects of the internet-based self-help intervention, we will conduct within-group analyses using repeated measures ANOVA (pre-intervention, post-intervention and follow-up measures) and paired *t* tests when comparing only two time points.

Moreover, we will exploratory analyze possible predictors, mediators, and moderators for the relationship between the internet-based self-help program and the outcomes. The significance level is set at 5%. Analyses will be conducted using SPSS and R.

### Interim analyses {21b}

Not applicable since no interim analyses are planned.

### Methods for additional analyses (e.g., subgroup analyses) {20b}

Not applicable since no additional analyses such as subgroup analyses are planned.

### Methods in analysis to handle protocol non-adherence and any statistical methods to handle missing data {20c}

Statistical analyses will be carried out according to the intention-to-treat approach and therefore will include all randomized participants. The extent of missing data will be analyzed. We will explore missing data patterns and determine the type of missing data (missing completely at random, missing at random, not missing at random). We will use multiple imputation to substitute missing values and will conduct sensitivity analyses for both the datasets with and without the imputed data.

### Plans to give access to the full protocol, participant level-data, and statistical code {31c}

There are no plans for granting public access to the full protocol, participant-level dataset, and statistical code.

## Oversight and monitoring

### Composition of the coordinating center and trial steering committee {5d}

There is no trial steering committee. The composition of the coordinating center is as follows:

- Principal investigator: HZ
  - ○ Design and conduct of the study
  - ○ Publication of study reports
  - ○ Preparation of protocol and revisions and case report forms
- Co-principal investigator: TB
  - ○ Design and conduct of the study
  - ○ Publication of study reports
  - ○ Preparation of protocol and revisions and case report forms
- PhD students: NB and JH
  - ○ Supporting the principal and co-principal Investigator in all the above responsibilities
  - ○ Data entry and management
  - ○ Recruitment of participants

### Composition of the data monitoring committee, its role and reporting structure {21a}

As to the best of our knowledge, the internet-based self-help program in itself does not bear risks for the participants. Therefore, a data monitoring committee is not required. The principal investigator, the co-principal investigator and the PhD students warrant for data and participant safety.

### Adverse event reporting and harms {22}

In this trial, adverse events are defined as unintended negative developments in the participants, which may occur at the time of the use of the internet-based self-help program, but do not have to be causally related to its use. Those unintended negative developments in the participants include acute suicidality and hospitalization. Such adverse events and the corresponding actions taken will be documented in the case report form.

### Frequency and plans for auditing trial conduct {23}

The research management of the Faculty of Human Sciences at the University of Bern, an independent research control unit, warrants the auditing. There will be on site monitoring visits on a regular basis. The monitoring visits are documented in a monitoring report form. The data monitoring committee controls study procedures such as the site progress and enrollment, obtaining participant informed consent, randomization, or the reporting of adverse events.

### Plans for communicating important protocol amendments to relevant parties (e.g., trial participants, ethical committees) {25}

Important protocol amendments will be reported to the relevant parties (i.e., the Cantonal Ethics committee Bern, the trial participants and trial registries) by e-mail. Substantial amendments are only implemented after approval of the Cantonal Ethics committee Bern. All non-substantial amendments are communicated to the Cantonal Ethics committee Bern within the Annual Safety Report.

### Dissemination plans {31a}

Trial participants and the general population are informed about the results of the study by means of a results report.

## Discussion

The internet-based self-help program ROCO is, to the best of our knowledge, one of the first internet-based self-help programs specifically developed for the treatment of psychological distress due to the situation surrounding the COVID-19 pandemic. The results will give insight into the efficacy and acceptance of an internet-based self-help program in the context of COVID-19 pandemic-related psychological distress. Moreover, the results will contribute to the further adaption of the self-help program. In light of possible multiple waves and future pandemics, it is important to investigate the effectiveness of such psychological interventions as mental health resources might be strained.

Limitations of this study include that only short-term effects of the internet-based self-help program can be determined, since the waiting control condition already receives access to the self-help program after 3 weeks.

## Trial status

Trial start date: May 2020.

Currently recruiting (*N* = 99, January 2021).

Approximate date when recruitment will be completed: April 2021.

Version 3: 28. January 2021.

## Data Availability

The principal investigator, the co-principal investigator and the PhD students have access to the full data sets. All data collected is stored on a firewall-encrypted back-upped server of the University of Bern with strictly regulated access only for researchers directly involved in the study.

## Abbreviations

AjD: Adjustment disorder
BEI: Bern Embitterment Inventory
CAU: Care as usual
CBT: Cognitive behavioral therapy
CD-RISC: Connor-Davidson Resilience Scale
COVID-19: Coronavirus-19
CSQ-8: Client Satisfaction Questionnaire-8
DASS-21: Depression Anxiety Stress Scale
GSE: General Self-Efficacy Scale
LOT-R: Life Orientation Test Revised
PHQ-9: Patient Health Questionnaire
RCT: Randomized controlled trial
SARS: Severe acute respiratory syndrome
SBQ-R: Suicide Behavior Questionnaire Revised
SEK-27: Self-report measure for the assessment of emotion regulation skills
SF-12: 12-Item Short-Form Health Survey
SUS: System Usability Scale
ULS: UCLA Loneliness Scale
